# Supporting clinical decision making in the emergency department for paediatric patients using machine learning: A scoping review protocol

**DOI:** 10.1371/journal.pone.0294231

**Published:** 2023-11-16

**Authors:** Fiona Leonard, Dympna O’Sullivan, John Gilligan, Nicola O’Shea, Michael J. Barrett

**Affiliations:** 1 School of Computer Science, Technological University Dublin, Dublin, Ireland; 2 Digital Health Department, Children’s Health Ireland, Crumlin, Dublin, Ireland; 3 Library and Information Service, Children’s Health Ireland at Crumlin, Dublin, Ireland; 4 Department of Paediatric Emergency Medicine, Children’s Health Ireland at Crumlin, Dublin, Ireland; 5 Women’s and Children’s Health, School of Medicine, University College Dublin, Dublin, Ireland; Universitas Pelita Harapan, INDONESIA

## Abstract

**Introduction:**

Machine learning as a clinical decision support system tool has the potential to assist clinicians who must make complex and accurate medical decisions in fast paced environments such as the emergency department. This paper presents a protocol for a scoping review, with the objective of summarising the existing research on machine learning clinical decision support system tools in the emergency department, focusing on models that can be used for paediatric patients, where a knowledge gap exists.

**Materials and methods:**

The methodology used will follow the scoping study framework of Arksey and O’Malley, along with other guidelines. Machine learning clinical decision support system tools for any outcome and population (paediatric/adult/mixed) for use in the emergency department will be included. Articles such as grey literature, letters, pre-prints, editorials, scoping/literature/narrative reviews, non-English full text papers, protocols, surveys, abstract or full text not available and models based on synthesised data will be excluded. Articles from the last five years will be included. Four databases will be searched: Medline (EBSCO), CINAHL (EBSCO), EMBASE and Cochrane Central. Independent reviewers will perform the screening in two sequential stages (stage 1: clinician expertise and stage 2: computer science expertise), disagreements will be resolved by discussion. Data relevant to the research question will be collected. Quantitative analysis will be performed to generate the results.

**Discussion:**

The study results will summarise the existing research on machine learning clinical decision support tools in the emergency department, focusing on models that can be used for paediatric patients. This holds the promise to identify opportunities to both incorporate models in clinical practice and to develop future models by utilising reviewers from diverse backgrounds and relevant expertise.

## Introduction

Machine learning (ML) can be applied to emergency department (ED) data to develop clinical decision support systems (CDSS) for paediatric patients, providing health professionals with objective criteria. With the abundance of medical data being collected in electronic healthcare record systems, each patient has become a ‘big data’ challenge. Problems that would have involved collective thinking from clinicians to solve have now become too complex, reaching beyond the ability of the human mind [1], directing us towards ML as a decision support tool, helping to provide patients with the best possible care [2].

Artificial Intelligence (AI) is an umbrella term that includes a wide range of sub fields such as robotics, natural language processing, expert systems, knowledge representation, computer vision, intelligent agents, navigation, planning and predictive analytics [3]. ML is one of these sub fields and the focus of this research. ML is a technique used to train computer systems to automatically learn from data and past experiences to identify patterns and make predictions without being given explicit instructions, mimicking human intelligence [4]. A CDSS is an application used to enhance healthcare delivery by incorporating specific clinical knowledge, patient data, and other health information into medical decisions. CDSSs can be categorised as knowledge based and non-knowledge based. Programmable rules (example IF-THEN statements) are used in knowledge-based systems, retrieving data to evaluate the rule and providing an output or action. Non-knowledge based systems apply AI or ML to the data source as opposed to being programmed [5]. Similar to how imaging technology enhances medical decision making, ML as a CDSS tool has the potential to assist with complex medical decisions [6] and operational management within an environment that needs fast and accurate decision making such as the ED. Research has shown that CDSSs have proven to support clinicians in a range of decisions and patient care activities, and today they actively and widely enhance the provision of high quality care [5]. However, it is believed that a computer cannot fully replace the essential and intimate relationship between a clinician and a patient, nor can an algorithm fully replace the capacity of human reasoning [7], therefore caution should be exercised to avoid overdependence and to use these tools to enhance decision-making in an appropriate manner.

The objective of the scoping review is to perform a search of the literature for ML models used as CDSS tools in the ED. The data relating to challenges with paediatric datasets, areas of application, composition and validation will be sought in the literature for paediatric ML CDSS tools for use in the ED. Models developed using adult or mixed patient data will also be included as there may be potential to apply these models to paediatric patients in the ED. It will also enable us to compare the amount of research devoted to the development of ML CDSS tools for adults compared to paediatrics in the ED. To our knowledge no scoping reviews exist that focuses solely on the application of ML in the ED for clinical decision support, which also gathers information specific to the development of ML CDSS tools for paediatric patients in the ED.

## Materials and methods

This protocol will outline the process for conducting the scoping review. The methodology followed will be informed by the scoping study framework of Arksey and O’Malley [8] consisting of six stages.

1. Identifying the research question
2. Identifying relevant studies
3. Study selection
4. Charting the data
5. Collating, summarising and reporting the results
6. Consultation

The Joanna Briggs Institute (JBI) guidelines for scoping reviews [9] will be used to supplement the approach providing additional guidance, enabling transparency and replication of the study by predefining objectives, methodology and how the scoping review will be reported. The Preferred Reporting Items for Systematic Reviews and Meta-Analyses-Scoping Review Extension (PRISMA-ScR) [10] will be used in conjunction with these approaches (S1 Appendix). This scoping review protocol was registered at Open Science Framework (https://osf.io/75gvs).

### 1. Identifying the research question

The research question is structured using the key elements of Population, Concept and Context (PCC) [9] and aims to discover:

“How does machine learning support clinical decision making in the emergency department for paediatric patients?”

Although the research question focuses on the application to a paediatric population, this scoping review seeks to broaden the search criteria beyond paediatric and mixed settings. Adult settings will be included as some ML CDSS tools may be transferable to paediatrics, for example operationally for resource management. Additionally, it will be used to compare the research effort for ML CDSS tools developed for adults versus paediatrics.

### 2. Identifying relevant studies

The JBI guidelines provide more detailed instructions for the inclusion criteria and search strategy and will therefore be followed for this stage.

#### Inclusion criteria

The PCC framework used to formulate both the title and research question will be used to assist in defining the inclusion criteria.

*Population*. Although the population of interest is paediatrics, the scoping review will include all age groups to identify any gaps and opportunities in the literature that can be applied to paediatric patients in ED settings and to gain an understanding of the extent of research literature for adults compared to paediatrics.

*Concept*. There are two key elements of the core concept: ML and CDSS. ML includes various types such as supervised, unsupervised, semi-supervised, reinforcement learning [11] and deep learning [12]. This review will include these ML types along with their application to natural language processing and computer vision. As many methods in both ML and statistics can be used to create prediction models [13], studies focusing purely on statistical inference will be excluded. The CDSS will include any outcome that is used to assist clinical decisions for either the patient or planning and improving operational efficiencies. The search criteria will not use the term ‘decision support’, its inclusion could potentially omit relevant articles as it may not always be explicitly stated.

*Context*. Studies that use ML in an ED setting for decision support will be included, therefore out of hospital settings such as ambulatory and Emergency Medical Service calling centres fall outside the criteria being sought. There is no limitation in hospital institution settings (Secondary, tertiary and quaternary level), country or health system.

*Types of evidence sources*. Grey literature, letters, editorials, pre-print articles, papers whose abstract or full text is unavailable will not be included as they may not contain enough information to make a meaningful contribution to the review. Studies that do not have the abstract or full text in English will be excluded based on no resources available to adequately search and translate. Based on the results retrieved from initial literature searches and adopting a similar approach as Boonstra and Laven [14], the search period will include articles from the last five years reflecting the most contemporary sample of the literature. Study designs such as systematic, scoping and narrative reviews will be excluded as this proposed scoping study will seek to include the original research. Also excluded will be studies using simulation models and those based purely on synthesised data. Although synthesised data is very useful to fill the gap in the paucity of medical data in real-world scenarios, this data can sometimes propagate bias based upon the sample data on which it is derived [15].

#### Search strategy

As recommended by the JBI guidelines a three step search strategy will be carried out [9]. The first step already carried out involved initial limited searches of Medline (EBSCO) and CINAHL (EBSCO) to identify relevant keywords in the titles and abstracts along with index terms describing articles of interest. The second step will use all keywords and index terms relevant to the research question to develop a search strategy for each database to be included in the review. The third step will consist of searching the reference list of identified articles for additional sources. The reference list will only be examined in articles where full-text screening has been performed. An experienced research librarian provided advice on the design and implementation of the search strategy, including the identification of keywords and indexes.

The databases to be searched include Medline (EBSCO), CINAHL (EBSCO), EMBASE and Cochrane Central. Keywords such as “Emergency Department”, “Triage”, “Machine learning”, “Artificial Intelligence”, “Deep Learning”, “Natural Language Processing”, “Computer Vision”, “Predict” and “Model” along with various synonyms were included in the search strategy, which will be adapted to include the relevant index terms for each database. In line with JBI guidelines, an example of the full search strategy for Medline (EBSCO) is included in the supporting information (S2 Appendix).

### 3. Study selection

Each database’s search results will be imported into Rayyan (https://www.rayyan.ai) [16] which will be used to eliminate any duplicates and assist with the selection and review process. At the start of the screening process Zotero will be used to identify and exclude retracted articles. The labels used to assist in the categorisation of study types and the reasons for exclusion of articles will be decided by the review team (based on the inclusion criteria) and will be set up in Rayyan. Following similar methodology of previous researchers that distributed the workload using teams of reviewers [17–19], teams consisting of two reviewers will independently screen each article for inclusion against the eligibility criteria using the title and abstract. Discussion between the reviewers will resolve any disagreements. The independent groups of researchers will then review the full text of the remaining articles using the same inclusion criteria and will also examine the reference lists of included articles for additional sources.

**Fig 1:**
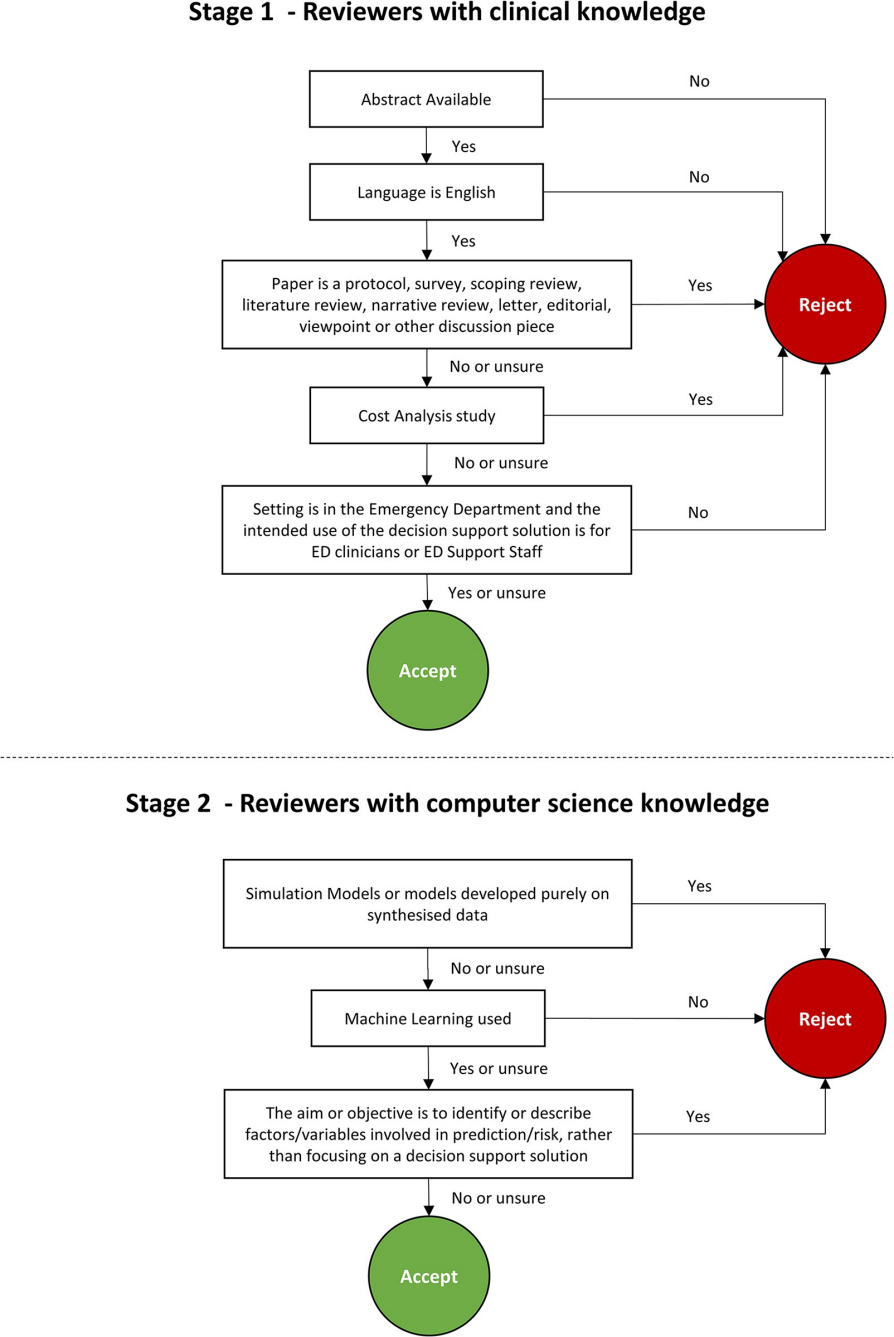
Article checklist used for title and abstract screening.

Several practice runs were performed by the review team, using between 25 and 100 randomly selected articles imported into Rayyan to ensure a robust screening process, inclusion criteria and to discuss how disagreements will be resolved. It was evident from these practice runs that the greatest source of disagreement originated from the reviewer’s background knowledge. The team consists of reviewers with strong clinical or computer science knowledge, therefore it was decided to leverage this knowledge by dividing the teams and the screening process into two stages. In the first stage, the reviewers with clinical knowledge will determine if the setting and the intended use of the CDSS tool is the ED. In the second stage, the reviewers with computer science knowledge will assess whether ML was used in the study to create a CDSS tool. For both stages, individual reviewers will be used to uphold the independent screening process. Following JBI guidelines [9], the full scoping review will not proceed until a 75% agreement or greater is achieved in the practice runs for both stage one and stage two. A checklist based on the PCC inclusion criteria (divided into the two stages) was created to assist the reviewers further in defining what should and should not be included (Fig 1). Using PRISMA-ScR guidelines a flow diagram representing the selection of sources of evidence will be included.

### 4. Charting the data

A preliminary charting table has been included which will contain key information relevant to the review question (Table 1). The Transparent Reporting of a multivariable prediction model for Individual Prognosis Or Diagnosis (TRIPOD) guidelines [20] were also used to inform the data items in the charting table, along with the approach taken by Andaur Navarro et al. [21]. As per the study selection stage the reviewers conducted a trial run using 100 articles in Rayyan to identify useful information to be included in the charting table. Additional items may be added to this table as the study progresses.

**Table 1:** Preliminary charting table.

Item	Description (with some examples of categories, which will be added to as the study progresses)
Author(s)	
Title	
Year of publication	
Journal	
Country	
Income	High-income, middle-income and low-income
Key study dates	
Hospital institution level	Secondary, tertiary, quaternary
Purpose	Diagnostic, prognostic, human v machine learning comparison
Decision support type	Patient or Planning/Operational
Clinical decision support user	Who the clinical decision support tool is intended to be used by (doctor, Triage nurse, bed manager, administrator)
Potential for paediatric use	Yes/No
Challenges addressed for implementing the model for paediatric patients	Yes/No/Not Applicable
If Yes, what were the challenges in implementing the model for paediatric patients	
Sample type	Paediatric, adult, mixed
Source of data	Retrospective, prospective, cohort, randomised trial, registry data
Sample size	
How was the sample size arrived at?	
Sampling type	Random sampling, cluster sampling, systematic sampling, stratified random sampling, selective sampling, bootstrapping, cross validation
How was missing data handled?	Complete-case analysis, single imputation, multiple imputation
Number of records excluded	
Data limitations	Nonrepresentative sample, few events per predictor, missing data).
Type of outcome	Discharge destination, triage category, returns, disease detection, fracture detection, number of visits…
Outcome aim	Classification, risk
Format of outcome variable	Binary, continuous, count, ordinal, multinominal, time-to-event
Dataset split type	Train:test, train:test:validation, temporal
Dataset split ratio	Example: 70:30, 50:25:25, January to June 2022:July to December 2022
Model development	Development, Development and External validation
Machine learning type	Logistic regression, linear regression, naïve Bayes, random forest, deep neural network, k nearest neighbour, mixed methods, ARIMA)
Type of machine learning method	Supervised, unsupervised, semi-supervised, reinforcement learning…
Hyperparameter optimisation	Grid search, manual search, cross-validation
Technical intervention used for challenges with paediatric dataset	Sampling for class imbalance (Undersampling, Oversampling), z-scores
Other technical operations performed	Natural language processing, computer vision, dimension reduction…
Type of predictors used	Types of predictors used in the machine learning algorithm, which will also provide information on the level of data maturity of the study/hospital
Feature engineering tasks performed	
Predictor selection	Stepwise, forward, backward, all
Number of events per predictor	
Number of predictors used	
External validation type	Geographical, different care setting, proven and implemented in clinical practice
External Validation: Number of Sites	Number of sites or centres used in external validation
Evaluation type	Area under the curve, F1 score, root mean squared error, accuracy, specificity, sensitivity
Variable Importance reported	Yes, No
Reporting guidelines used	TRIPOD, STROBE, None mentioned…
Model availability	Repository for data, repository for code, model available (e.g. online form, simplified scoring rule)

### 5. Collating, summarising and reporting the results

Simple frequency counts and percentages of items collected in the charting table will be performed, as the focus of this scoping review is quantitative analysis. A combination of charts and tables will be used to present the data and will be designed using Microsoft excel and R Studio. Data will be grouped into the general characteristics of the study design, the elements of the PCC to align with the objectives of the scoping review, study types, characteristics, any methods used to overcome challenges with paediatric data, technical methodology, implementation in practice, variables used to develop the ML models, evaluation and validation. These groupings will be used to find potential gaps in the literature and potentially identify which topics demand further investigation. The list and occurrence of variables used in each study design will assist in determining how reproducible these studies can be in countries where these data points are not readily available in an electronic format. The method of presentation of the results will be further refined during the review process.

### 6. Consultation

Stakeholder engagement will involve paediatric emergency medicine teams in Children’s Health Ireland and the research collaborative: Paediatric Emergency Research in the United Kingdom and Ireland (PERUKI).

The list of articles retrieved from the database searches will be made publicly available. This result set will include an indication of the articles that were excluded during the screening process.

## Discussion

Previous literature reviews have broadened the scope beyond the ED to include emergency medicine, which covered pre-hospital settings (e.g. paramedics, emergency medical services) [4, 22], others narrowed it by focusing on particular areas such as triage [23, 24], predicting in-hospital admissions [25] and others that excluded some application areas of planning and operational management [4]. Several reviews only included studies that had evidence that the model outperformed accepted practices [14] or in the study by Kareemi et al., manuscripts were selected based on evaluating the performance of the models against standard care for prognosis and diagnosis prediction [26]. When looking at the intended users of these CDSS tools, Boonstra and Laven restricted their research to use by ED physicians [14]. Several researchers excluded paediatric studies from their reviews [23, 26]. Medical databases were predominately used in the search, with some authors including technology databases [22]. Liu et al. opted to include one clinical trials database when searching for registered trials on AI in the ED [27]. Many researchers included all AI applications [4, 14, 22] as opposed to ML, with some excluding research that used classical methods such as logistic regression if not a validated model [26] and others excluding studies that did not self-identify as AI [22]. Four reviews were identified as encompassing some of the aspects that this scoping review will use in the search strategy with some potential gaps (Table 2). Liu at al. also carried out a review of AI in emergency medicine [28], but there was no evidence that a scoping or systematic review was carried out.

**Table 2:** Pre-existing scoping and systematic literature reviews.

Author	Year of Publication	Dates included in Search	Aim	Potential Gaps
Boonstra and Laven [14]	2022	2017 to 21^st^ April 2021	Aimed to show how artificial intelligence is currently applied in emergency departments and how this affects the way emergency department clinicians approach their work.	Focuses on artificial intelligence for use by emergency department physicians only. Excluded any study that had a negative outcome and did not outperform accepted practices. An analysis of the different kinds of artificial intelligence based tools used in the studies was not addressed.
Kareemi et al. [26]	2021	Date of inception to 17^th^ Oct 2019	Aimed to determine whether machine learning models performed better than standard care in predicting diagnosis and prognosis for patients in emergency departments.	Focused study that only included papers that compared a machine learning model against a standard of care approach for prognostic or diagnostic prediction. Excluded research involving only paediatric patients. Logistic regression was excluded unless part of a prediction model that was validated.
Tang et al. [4]	2021	Between the years 2014 and 2020.	Aimed to provide an overview of the uses of artificial intelligence for emergency medicine by examining current advancements in the management of emergency departments and in patient care.	Broader scope of all emergency medicine. Limited the focus to four main applications of machine learning in emergency medicine. These applications included 1. Pre-hospital 2. Emergency department management 3. Triage, disposition and patient acuity 4. Prediction of medical conditions/ailments.
Kirubarajan A, Taher A, Khan S, et al. [22]	2020	Articles published up to 28^th^ February 2020	Aimed to locate available literature on artificial intelligence applications in emergency medicine.	Broader scope of emergency medicine, which included out of hospital settings. Studies that did not explicitly self-identify as Artificial Intelligence in the titles or abstract may have been missed by the selected search approach.

There are several strengths to consider. This scoping review will be the first to focus on ML CDSS tools for use in the ED, that will also seek to understand where the development of paediatric specific ML CDSS tools lies within that research landscape. As this scoping review will include reviewers from diverse backgrounds and expertise, a different approach will be taken in the screening process to leverage this knowledge appropriately. Splitting the review teams in the early stage of the screening process into those with clinical knowledge (stage 1) and computer science knowledge (stage 2) will decrease the time spent on resolving disagreements that may arise due to lack of either clinical or ML knowledge. During a trial run involving the screening of 100 articles, this difference in backgrounds and area of expertise proved to be one of the top reasons for disagreements on literature to include in the review.

Limitations of this study include the exclusion of literature whose abstract or full text is not in English. Studies using entirely synthesised data or simulated models have also been excluded as this scoping review seeks to capture data on the evaluation and validation methods of models using real world data. The databases to be included in the search are predominately medical based, therefore some useful research may be missed from other types of databases such as computer science.

To conclude, this scoping review will be an important source of information for future researchers looking to develop ML CDSS tools for paediatric patients in the ED.

## Supporting information

S1 Appendix PRISMA-ScR checklist. Preferred Reporting Items for Systematic reviews and Meta-Analyses extension for Scoping Reviews (PRISMA-ScR) Checklist.
(PDF)

S2 Appendix Search strategy for Medline (EBSCO). (PDF)
