# Supplementary material for: Supporting clinical decision making in the emergency department for paediatric patients using machine learning: A scoping review protocol
Source: PLoS One. 2023 Nov 16;18(11):e0294231. doi: 10.1371/journal.pone.0294231 (PMC10653406; doi:10.1371/journal.pone.0294231)
Supplement: S2 Appendix — (PDF) [file pone.0294231.s002.pdf]

S2 Appendix. Search strategy

Medline (EBSCO)

No.	Query	Limiters/Expanders
S3	S1 AND S2	Limiters - Date of Publication: 20180101-20221231
		Expanders - Apply equivalent subjects
		Search modes - Boolean/Phrase
S2	AB (("triage" N1 ("centre*" OR "center*" OR "department*" OR "unit*")) OR ("accident and emergency") OR ("accident & emergency") OR ("urgent care") OR (emergency N1 (room* OR department* OR service* OR unit* OR visit* OR attendance* OR treatment* OR care)) OR (casualty N1 (department* OR room)) OR (trauma N1 (centre* OR center* OR care))) OR<br><br>TI (("triage" N1 ("centre*" OR "center*" OR "department*" OR "unit*")) OR ("accident and emergency") OR ("accident & emergency") OR ("urgent care") OR (emergency N1 (room* OR department* OR service* OR unit* OR visit* OR attendance* OR treatment* OR care)) OR (casualty N1 (department* OR room*)) OR (trauma N1 (centre* OR center* OR care))) OR<br><br>(MH(("Emergency Service, Hospital+") OR ("Emergency Medical Services") OR ("Triage")))	Expanders - Apply equivalent subjects<br><br>Search modes - Boolean/Phrase
S1	(AB(("predict*" N5 "model*") OR ("machine learning") OR ("artificial intelligence") OR ("deep learning") OR ("natural language processing") OR ("image recognition") OR ("computer processing") OR ("computer vision"))) OR<br><br>(TI(("predict*" N5 "model*") OR ("machine learning") OR ("artificial intelligence") OR ("deep learning") OR ("natural language processing") OR ("image recognition") OR ("computer processing") OR ("computer vision"))) OR<br><br>(MH(("Machine Learning+") OR ("Artificial Intelligence+") OR ("Image Processing, Computer-Assisted")))	Expanders - Apply equivalent subjects<br><br>Search modes - Boolean/Phrase
